# Find the Essence through the Phenomena: Cardiovascular Diseases and Biomarkers

**DOI:** 10.1155/2018/1929106

**Published:** 2018-06-24

**Authors:** Zhongjie Shi, Agata M. Bielecka-Dabrowa, Ospan A. Mynbaev, Shipeng Wei

**Affiliations:** ^1^Wayne State University, Detroit, MI, USA; ^2^Medical University of Lodz, Lodz, Poland; ^3^Moscow Institute of Physics and Technology, Moscow, Russia; ^4^Department of Internal Medicine, Augusta University, Augusta, USA

Biomarkers are often measured and evaluated to determine normal biological processes, pathological processes, or pharmacological responses to a therapeutic intervention. Our current issue focuses on new advances in cardiovascular diseases and biomarkers. The goal of this special issue is to summarize new biomarkers in risk prediction, screening, diagnosis, progression, and prognosis of cardiovascular diseases; measurement relevance of biomarkers addressing specificity, sensitivity, and biological variation of these biomarkers; and the role of biomarkers in evaluation and guidance of drug therapies. The importance of these biomarkers has been raised to the level that they have never been to before. Not simply because more research work has been done, and also because people realize that they can provide some information that could not be obtained by other means.

J. Tian et al. reported prognostic association of circulating neutrophil count with no-reflow in patients with ST-segment elevation myocardial infarction (STEMI) following successful primary percutaneous coronary intervention (PCI). A circulating neutrophil count ≥ 9.14 ×  10^9^/L is independently associated with no-reflow in patients with acute STEMI following primary PCI. J. Budzianowski et al. further discussed the importance of hematological indices in patients with acute coronary syndrome (ACS), including white blood cells (WBC), neutrophil to lymphocyte ratio (NLR), red cell distribution width (RDW), and platelet indices, such as platelet to lymphocyte ratio (PLR), mean platelet volume (MPV), and platelet distribution width (PDW) in the setting of ACS. M. Rabajdová et al. reported the detection of pathological changes in the aorta during thoracic aortic aneurysm progression. It is significantly associated with increased mRNA and protein levels of inflammatory cytokines (CRP and IL-6). A. Caraba et al. reported vitamin D status, disease activity, and endothelial dysfunction in early rheumatoid arthritis (RA) patients. They found that in early RA patients with moderate and high disease activity, low serum level of vitamin D is associated with disease activity, increased insulin resistance, and endothelial dysfunction. A. Kolaszko et al. reported the role of parathyroid hormone (PTH) and vitamin D serum concentrations in patients with cardiovascular diseases. They found that PTH serum concentration in contrast to 25-hydroxyvitamin D (25(OH)D), but not phosphorus, and Ca^2+^, significantly elevates among the patients with heart failure and shows significant correlation with their clinical status expressed by the New York Heart Association (NYHA) classification. T. Chen et al. reported a correlation between serum gamma-glutamyl transferase (GGT), serum ferritin (SF), and the rate of CKD and found that GGT and SF synergistically influence the rate of CKD. In other words, patients with abnormal SF and GGT may have higher risk to develop cardiovascular diseases.

Other serum markers, like troponin, N-terminal pro-brain natriuretic peptide (NT-proBNP), and apelin, are relatively new markers coming to the cardiovascular field, and more research work came out since then. R. Rajtar-Salwa et al. found that elevated troponin, but not NT-proBNP, is associated with increased risk of sudden cardiac death in hypertrophic cardiomyopathy (HCM) patients. Salska et al. also reported that pro-brain natriuretic peptide (pro-BNP) is not a marker of arrhythmia recurrence, whereas higher apelin concentration at admission indicates ineffectiveness of direct-current cardioversion or recurrence of arrhythmia within a month.

Gene polymorphism and mutation is gaining more and more spotlights nowadays. H. Song et al. found that interleukin-31 gene polymorphisms are tightly associated with dilated cardiomyopathy (DCM) susceptibility and contributes to worse prognosis in DCM patients in a Chinese population. J. Li et al. reported the protective role of cullin-3 single-nucleotide polymorphism rs17479770 in essential hypertension in male Chinese Han population. S. Sirotina et al. reported that a novel polymorphism in the promoter of the CYP4A11 gene is associated with susceptibility to coronary artery disease (CAD). M. A. Sazonova et al. reported that mitochondrial genome mutations are associated with myocardial infarction. It could be foreseen that in the future, gene screening will make treatment more individualized, such as early primary prophylaxis and more aggressive treatment as secondary prophylaxis for gene-positive patients.

Looking back at what has been accomplished by our colleagues before us and looking ahead what we need to accomplish to get our research to the next level, we believe that we will find more through the surface and eventually get to the core of it, the essence of science.

*Zhongjie Shi* *Agata M. Bielecka-Dabrowa* *Ospan A. Mynbaev* *Shipeng Wei*

